# Prevalence of ERG expression and PTEN loss in a Brazilian prostate cancer cohort

**DOI:** 10.1590/1414-431X20198483

**Published:** 2019-12-05

**Authors:** C.E. Morais, D.C. Gurgel, A.C. Teixeira, T.V. Arruda Mattos, A.V. Alves da Silva, F. Tavora

**Affiliations:** 1 Laboratório Argos Patologia, Fortaleza, CE, Brasil; 2 Departamento de Patologia, Universidade Federal do Ceará, Fortaleza, CE, Brasil; 3 Departamento de Anatomia Patológica, Santa Casa Misericórdia de São Paulo, São Paulo, SP, Brasil; 4 Centro Universitário Christus (Unichristus), Fortaleza, CE, Brasil

**Keywords:** ERG expression, PTEN, Biomarker, Radical prostatectomy, Prostatic carcinoma

## Abstract

*PTEN* is the most commonly inactivated tumor suppressor gene in primary prostate cancer (PCa) and its loss is associated with poor clinical outcomes. *ERG* rearrangement is a genomic alteration frequently found in PCa and its prognostic significance has yielded mixed results. Although the association of PTEN and ERG biomarkers has potential impact on clinical outcomes, studies examining the two genes simultaneously are scarce in Brazilian populations. In this study, we retrospectively examined the relationship between *ERG* expression and *PTEN* loss in 119 surgically treated prostate cancer patients from Northeastern Brazil through immunohistochemical analysis. ERG expression was found in 41.0% (48/117) of cases and the loss of *PTEN* detected in 38.1% (40/105) of samples. ERG-positive cases were significantly associated with lower prostate weight; *ERG* negatively correlated with Gleason score above 6. The lack of associations for *PTEN* loss alone in this cohort is counter to the literature, which shows that *PTEN* loss is usually associated with more aggressive disease. The overlapping of the two biomarkers revealed that samples with positive ERG expression without PTEN loss were associated with lower Gleason and lower Grade group. This study contributes with the discussion about the development of the molecular profiling of prostate cancer. The further development of similar studies could help in stratifying specific risk groups, leading to a more personalized therapeutic decision for prostate cancer treatment.

## Introduction

The burden of prostate cancer (PCa) on male health is evident as it is one of the leading causes of cancer-related death among men worldwide (1). In Brazil, 14,500 deaths by PCa are registered annually and 68,220 new cases were estimated in 2018 (2). These numbers highlight the need for novel treatment approaches and prevention of PCa.

Although Gleason score system, serum prostate-specific antigen (PSA), and clinical and pathological tumor stage are established tools for grading PCa, they are not capable of distinguishing men with high risk disease from the indolent majority. As a result, patients on active surveillance may lose the correct timing for treatment or suffer from unnecessary side effects of overtreatment (3). To overcome these issues, a lot of approaches are being explored, such as molecular biomarkers aiming to give information about the tumor biology and help predict the course of the disease. Among these, phosphatase tensin homolog (PTEN), an oncogene suppressor, and TMPRSS2-ERG, a gene rearrangement, are some of the most studied.

PTEN is a tumor suppressor gene commonly inactivated in PCa. Its inactivation rate depends on the method used to assess its status and varies between 10 and 61% (4). PTEN loss has been associated with poor prognostic parameters such as higher Gleason score, advanced pathological stage (5–7), biochemical recurrence, shortened post-surgical disease-specific survival, shortened metastasis-free survival after radical prostatectomy, and disease-specific survival in castration-resistant PCa (8,9). PTEN loss is related to PI3K/mTOR activation, a pathway that could be druggable for developing a potential targeted therapy, as in other malignancies such as lung cancer (10,11).

The fusion between ETS-related gene (ERG) and androgen-regulated transcription factor, TMPRSS2, is the most prevalent genomic alteration in PCa, occurring in approximately half of PCa cases (12,13). This rearrangement has been associated to a poor prognosis (14,15) and could be a potential biomarker for neoplastic lesions such as high-grade prostatic intraepithelial neoplasia (HGPIN) and adenocarcinomas (16,17).

The characterization of molecular subtypes could be useful in understanding the differences in the course of PCa. However, the frequency of those subtypes varies among races adding more complexity to the understanding of the disease (18,19). Knowing the frequencies of these alterations and how they are involved with each other will provide valuable insights about the pathobiology of PCa, a knowledge that could be translated into better clinical practice.

In this study, we examined the expression of ERG and the loss of PTEN in 119 PCa patients from a large pathology laboratory in Northeastern Brazil and investigated the association between these biomarkers and clinicopathological features of the cases.

## Material and Methods

### Clinical sample selection

After approval by the Unichristus Ethics Committee (CAAE 61472016.3.0000.5049), a total of 97 radical prostatectomies (RP) with the diagnosis of usual acinar adenocarcinoma, performed between 2013 and 2016 and totally sampled in paraffin blocks, were randomly selected from the files of a reference pathology laboratory in the city of Fortaleza. Available clinical information and histopathological reports were reviewed. To enrich the study cohort, subjects with higher Gleason scores (Gleason ≥8) from 22 transurethral resections of the prostate (TURP) were also included in the study. No patient had history of neoadjuvant treatment. Cases with diagnosis of ductal adenocarcinoma and small cell neuroendocrine carcinoma were not included. All hematoxylin and eosin (HE) slides were reevaluated by 2 pathologists (CLM and FT) for choosing index tumor nodules in RP and the highest Gleason score foci in TURP for tissue microarray (TMA) construction. All clinicopathological information was obtained from the medical requisition forms.

### TMA construction

Two punches (2 mm cores) were taken from 8 paraffin-embedded tissue blocks from the dominant tumor nodule in RP and from tumor foci in TURP, sampled in duplicate. Benign prostate tissue from other RPs not included in this study were placed on the corner of each TMA to be used as external control.

### Immunohistochemical analysis

Two 4-mm consecutive sections were cut from each TMA block and mounted on charged microscopy glass slides for immunohistochemical staining for PTEN and ERG protein expression.

PTEN immunohistochemistry (IHC) was performed on the Ventana BenchMark^®^ platform (Ventana Medical Systems, USA) using rabbit monoclonal primary anti-PTEN antibody (Clone SP218; Roche Diagnostics, UK). PTEN protein status was visually scored. A tissue core was considered to have PTEN protein loss if the intensity of cytoplasmic and nuclear staining was markedly decreased or entirely negative across >10% of tumor cells compared to surrounding benign glands and/or stroma, which provided internal positive controls. If PTEN was lost in >10 and <100% of the tumor cells sampled in a given core, the core was annotated as showing heterogeneous PTEN loss (20). Alternatively, if the core showed PTEN loss in 100% of sampled tumor glands, the core was annotated as showing homogeneous PTEN loss. From 119 samples used in our study, 14 were excluded for technical problems that made it impossible to evaluate the marker (cores fell out from the TMA, no tumor sample, and difficulty in determining the score due to strong background staining). For statistical analysis, each patient's tumor sample was scored for PTEN loss by summarizing the scores of the sampled cores.

ERG IHC was performed on the BenchMark ULTRA automated IHC/ISH staining platform (Ventana Medical Systems) using a rabbit monoclonal anti-ERG antibody (Clone EP111, USA). ERG protein status was visually determined and scored as positive if any tumor cells demonstrated nuclear ERG staining. Endothelial cells were used as internal controls. The same criteria as PTEN was conducted for ERG, with samples for the presence or absence of ERG expression.

### Statistical analysis

Statistical analyses were performed using GraphPad Prism version 6.0 (GraphPad, USA). Correlations between categorical data variables were analyzed using Fisher's exact test or Person's chi-squared test. Quantitative variables with parametric distribution were analyzed using Student's *t*-test and analysis of variance (ANOVA), and quantitative variables with non-parametric distribution were analyzed using Kruskal-Wallis and Mann Whitney tests. For all analyses, a P value <0.05 was considered to be statistically significant.

## Results

The study comprised 119 subjects, including 97 (82%) RP and 22 (18%) TURP specimens. The mean age was 71 years (range 51-98 years). Only 13 patients had pre-treatment PSA serum levels, with a median of 8.5 ng/mL, 31 (26%) showed a Gleason score ≤6, 50 (42%) had a Gleason score of 7, and 38 (32%) had a Gleason score ≥8. When using prognostic grade groups (GG) (21), 31 (26%) were in GG1, 29 (24%) in GG2, 21 (18%) in GG3, 12 (10%) in GG4, and 26 (22%) in GG5.

Median prostate weight and tumor volume were 45 g and 20%, respectively. Extraprostatic extension, HGPIN, and seminal vesicle involvement were observed in 35 (36%), 86 (89%), and 8 (8%) cases, respectively. Final pathologic staging classified 57 (59%) cases as T2 N0/Nx and 40 (41%) as T3 N0/N1/Nx (Table 1).

**Table 1:** Clinicopathological characteristics of patients treated with radical prostatectomy or transurethral resection of the prostate (TURP) for prostate cancer.

Characteristics	n	
Age at diagnosis, years (mean, range)	115	71 (51–99)
PSA, ng/mL [median (q1-q3)]	13	8.5 (6.3–18.5)
Gleason score, n (%)		
Gleason 6		31 (26)
Gleason 7		50 (42)
Gleason ≥8		38 (32)
Grade group, n (%)	119	
Group 1		31 (26)
Group 2		29 (24)
Group 3		21 (18)
Group 4		12 (10)
Group 5		26 (22)
Type of procedure, n (%)	119	
Radical prostatectomy		97 (82)
TURP		22 (18)
Prostate weight, g [median (q1-q3)]	96	45 (35–55)
Tumor volume, % [median (q1-q3)]	97	20 (10–25)
Extraprostatic extension, n (%)	96	
Absent		61 (64)
Present		35 (36)
High-grade prostate intraepithelial neoplasia, n (%)	97	
Absent		11 (11)
Present		86 (89)
Seminal vesicles involvement, n (%)	97	
Absent		89 (92)
Present		8 (8)
Surgical margins, n (%)	97	
Negative		63 (65)
Positive		34 (35)
Lymphovascular invasion, n (%)	97	
Absent		92 (95)
Present		5 (5)
Staging, n (%)	97	
T2 N0/NX		57 (59)
T3 N0/N1/NX		40 (41)

PSA: prostate specific antigen.

**Figure 1:**
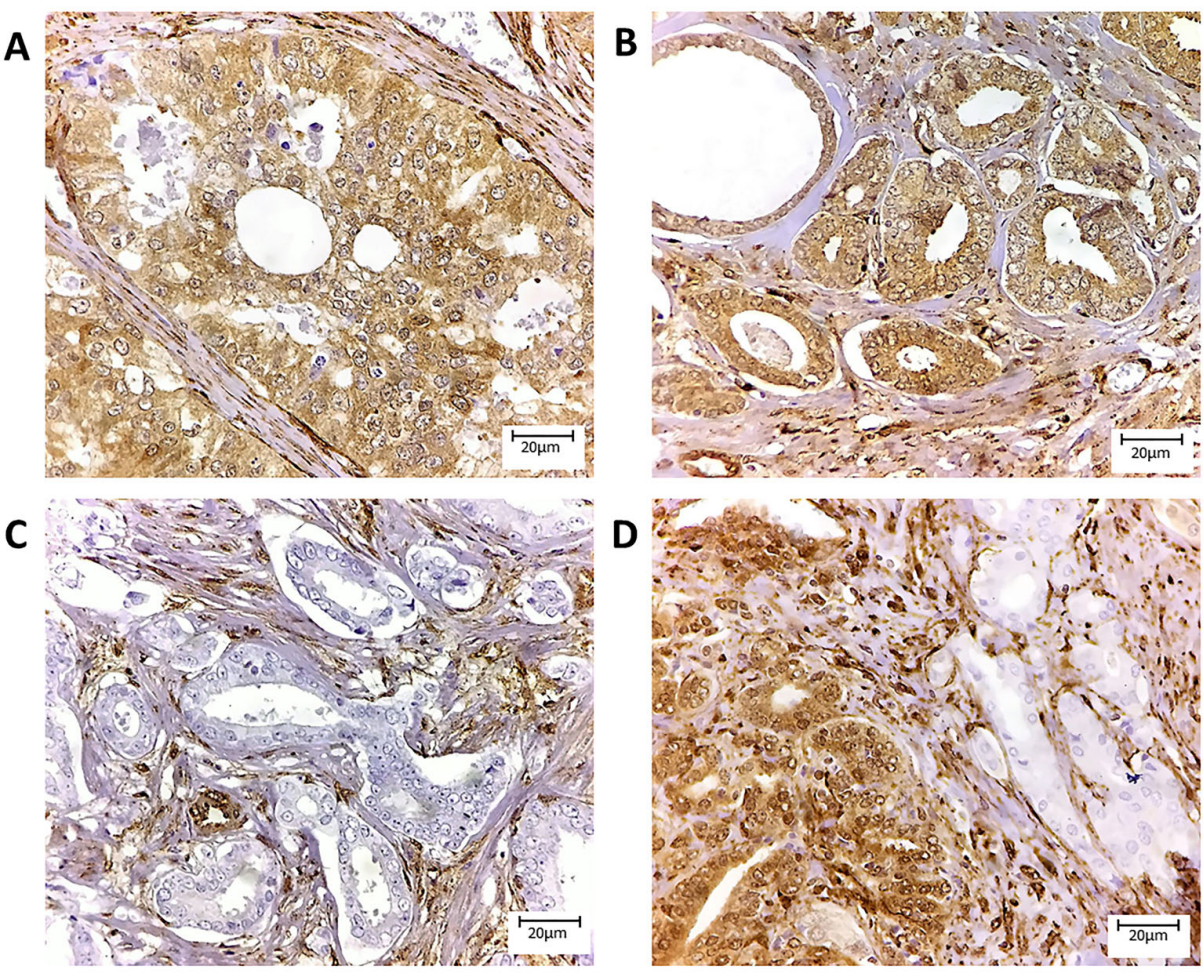
PTEN immunohistochemistry in prostate tissue showing several staining patterns on tissue microarray sections. **A**, Cribriform pattern with Gleason score 4+4 and positive cytoplasmic expression. **B**, Loose glands with Gleason Score 3+3 and cytoplasmic expression. **C**, Homogeneous PTEN loss on a Gleason score 3+3 tumor. **D**, Heterogeneous PTEN loss on Gleason Score 3+4 prostate tumor. Magnification bar: 20 μm.

**Figure 2:**
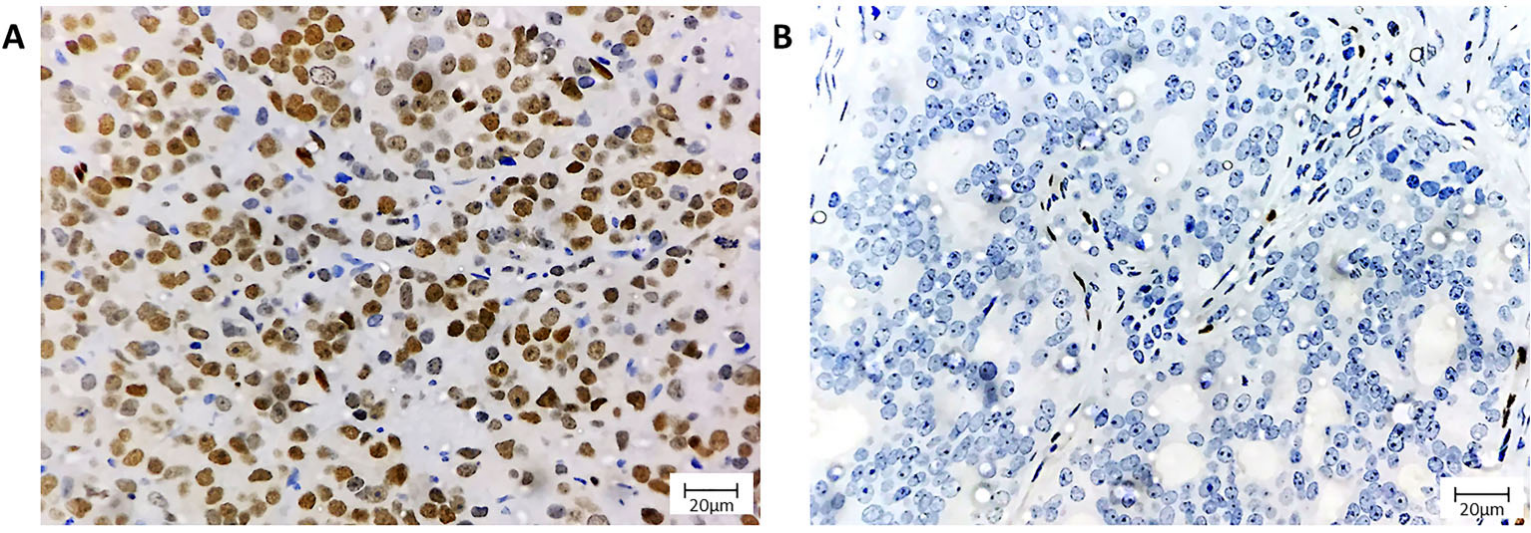
ETS-related gene (ERG) immunohistochemistry. **A**, Nuclear ERG positivity in prostate cancer. **B**, Negative expression in prostate cancer with positive nuclear internal control in endothelial cells. Magnification bar: 20 μm.

ERG was positive in 41% (48/117) of samples and was significantly associated with lower prostate weight (P=0.008) and Gleason score (P=0.040). There were no other important associations between ERG and the other clinicopathological features as shown in Table 2. PTEN loss was observed in 38% (40/105) of cases. Half of them showed heterogeneous loss pattern (20/40) (Figures 1 and 2). No associations with all clinicopathological features were demonstrated as shown in Table 3.

**Table 2:** Association of ETS-related gene (ERG) expression status and clinicopathological characteristics of men treated with radical prostatectomy or transurethral resection of the prostate (TURP) for prostate cancer.

Characteristics	n	ERG negative	ERG positive	P value
Age at diagnosis, mean in years (n)	113	72 (66)	71 (47)	0.4915
Gleason score, n (%)	117			0.0316^a^
Gleason 6		13 (19)	17 (35)	
Gleason 7		36 (52)	14 (29)	
Gleason ≥8		20 (29)	17 (35)	
Grade group, n (%)	117			0.0723
Group 1		13 (19)	17 (35)	
Group 2		19 (28)	10 (21)	
Group 3		17 (25)	4 (8)	
Group 4		5 (7)	6 (13)	
Group 5		15 (22)	11 (23)	
Type of procedure, n (%)	117			0.3491
Radical prostatectomy		58 (84)	37 (77)	
TURP		11 (16)	11 (23)	
Prostate weight, median in g (n)	94	50 (58)	40 (36)	0.0007^b^
Tumor volume, median in % (n)	95	20 (58)	20 (37)	0.2772
Extraprostatic extension, n (%)	94			1.000
Absent		36 (63)	24 (65)	
Present		21 (37)	13 (35)	
High-grade prostate intraepithelial neoplasia, n (%)	95			0.7355
Absent		7 (12)	3 (8)	
Present		51 (88)	34 (92)	
Seminal vesicles involvement, n (%)	95			0.1441
Absent		51 (88)	36 (97)	
Present		7 (12)	1 (3)	
Surgical margins, n (%)	95			0.6636
Negative		36 (62)	25 (68)	
Positive		22 (38)	12 (32)	
Lymphovascular invasion, n (%)	95			1.000
Absent		55 (95)	35 (95)	
Present		3 (5)	2 (5)	
Staging, n (%)	95			1.000
T2 N0/NX		34 (59)	22 (59)	
T3 N0/N1/NX		24 (41)	15 (41)	

Data are reported as number with pecent in parentheses. PSA: prostate specific antigen. ^a^Pearson's chi-squared test; ^b^Mann-Whitney U test.

**Table 3:** Association of measured phosphatase tensin homolog (PTEN) and clinicopathological characteristics of men treated with radical prostatectomy or transurethral resection of the prostate (TURP) for prostate cancer.

Characteristics	n	ERG negative	ERG positive	P value
Age at diagnosis, mean in years (n)	102	72 (39)	71 (63)	0.8629
Gleason score, n (%)	105			0.1965
Gleason 6		6 (15)	20 (31)	
Gleason 7		20 (50)	27 (42)	
Gleason ≥8		14 (35)	18 (28)	
Grade group, n (%)	105			0.1763
Group 1		6 (15)	20 (31)	
Group 2		13 (33)	13 (20)	
Group 3		7 (18)	14 (22)	
Group 4		6 (15)	4 (6)	
Group 5		8 (20)	14 (22)	
Type of procedure, n (%)	105			0.3491
Radical prostatectomy, n (%)		34 (85)	54 (83)	
TURP		6 (15)	11 (17)	
Prostate weight, median in g (n)	87	45 (33)	40 (54)	0.479
Tumor volume, median in % (n)	88	25 (34)	20 (54)	0.0659
Extraprostatic extension, n (%)	88			0.1155
Absent		17 (50)	37 (69)	
Present		17 (50)	17 (31)	
High-grade prostate intraepithelial neoplasia, n (%)	88			0.7355
Absent		4 (12)	6 (11)	
Present		30 (88)	48 (89)	
Seminal vesicles involvement, n (%)	88			0.4224
Absent		30 (88)	51 (94)	
Present		4 (12)	3 (6)	
Surgical margins, n (%)	88			0.2573
Negative		25 (74)	33 (61)	
Positive		9 (26)	21 (39)	
Lymphovascular invasion, n (%)	88			0.0773
Absent		30 (88)	53 (98)	
Present		4 (12)	1 (2)	
Staging, n (%)	95			1.000
T2 N0/NX		15 (44)	35 (65)	
T3 N0/N1/NX		19 (56)	19 (35)	

PSA: prostate specific antigen; TURP: transurethral resection of the prostate.

### ERG and PTEN associations

When PTEN and ERG were analyzed in conjunction (Table 4), PTEN loss was observed in 32% (20/63) of samples without expression of ERG protein, while among samples expressing ERG, PTEN loss was observed in 48% (19/40). However, the augmented frequency of PTEN loss among ERG-positive samples was not significant (P=0.1447) (Table 4)

**Table 4:** Association of PTEN and ERG expression overlapping with the clinicopathological characteristics of men treated with radical prostatectomy or transurethral resection of the prostate (TURP) for prostate cancer.

Characteristic	n	ERG-/PTEN-	ERG-/PTEN+	ERG+/PTEN-	ERG+/PTEN+	P value
Age at diagnosis, mean in years (n)	100	72 (19)	72 (41)	71 (19)	69 (21)	0.639
PSA, median in ng/mL (n)	12	8 (4)	7 (4)	17 (1)	14 (3)	-
Gleason score, n (%)	103					0.0131^a^
Gleason 6		2 (10)	8 (19)	4 (21)	11 (52)	
Gleason 7		13 (65)	23 (53)	7 (37)	4 (19)	
Gleason ≥8		5 (25)	12 (28)	8 (42)	6 (29)	
Grade group, n (%)	103					0.0369^a^
Group 1		2 (10)	8 (19)	4 (21)	11 (52)	
Group 2		7 (35)	12 (28)	6 (32)	1 (5)	
Group 3		6 (30)	11 (26)	1 (5)	3 (14)	
Group 4		1 (5)	3 (7)	4 (21)	1 (5)	
Group 5		4 (20)	9 (21)	4 (21)	5 (24)	
Type of procedure, n (%)	103					0.9866
Radical prostatectomy		17 (85)	36 (84)	16 (84)	17 (81)	
TURP		3 (15)	7 (16)	3 (16)	4 (9)	
Prostate weight, median in g (n)	85	55 (17)	49 (36)	44 (15)	37 (17)	0.0123^b^
Tumor volume, median in % (n)	86	24 (17)	20 (36)	22 (16)	17 (17)	0.2608
Extraprostatic extension, n (%)	86					0.1889
Absent		7 (41)	26 (72)	10 (63)	10 (59)	
Present		10 (59)	10 (28)	6 (38)	7 (41)	
High-grade prostate intraepithelial neoplasia, n (%)	86					0.9442
Absent		2 (12)	4 (11)	1 (6)	2 (12)	
Present		15 (88)	32 (89)	15 (94)	15 (88)	
Seminal vesicles involvement, n (%)	86					0.0646
Absent		13 (76)	34 (94)	16 (100)	16 (94)	
Present		4 (24)	2 (6)	0 (0)	1 (6)	
Surgical margins, n (%)	86					0.6909
Negative		12 (71)	22 (61)	12 (75)	10 (59)	
Positive		5 (29)	14 (39)	4 (25)	7 (41)	
Lymphovascular invasion, n (%)	86					0.255
Absent		15 (88)	35 (97)	14 (88)	17 (100)	
Present		2 (12)	1 (3)	2 (12)	0 (0)	
Staging n, (%)	86					0.3669
T2 N0/NX		7 (41)	23 (64)	8 (50)	11 (65)	
T3 N0/N1/NX		10 (59)	13 (36)	8 (50)	6 (35)	

PSA: prostate specific antigen. ^a^Pearson's chi-squared test; ^b^Kruskal-Wallis H test.

When all possible combinations between ERG and PTEN were considered, it was observed that a positive ERG expression overlapping with intact PTEN (ERG+/PTEN+) were significantly associated with lower Gleason scores (P=0.0131) and lower prognostic groups (P=0.0369). Prostate weight also showed statistical significance (P=0.0123) with ERG/PTEN profiling, demonstrating significantly lower weight in the ERG+/PTEN+ group.

## Discussion

Despite the progress in proteomic, genomic, and other omics data we have experienced over the last decades, PCa treatment is still guided mainly by pathologic grade, final stage, and serum PSA. The absence of specific biomarkers solely capable of providing information on the overall PCa outcome makes distinction between patients with aggressive and indolent disease challenging.

In addition, considering the relevance of race/ethnicity to the outcome of the disease, we studied the utility of two biomarkers, PTEN and ERG, in a population of PCa patients from Northeastern Brazil, a diverse geographical region with no previous study. In the present study, we evaluated ERG by IHC, a method that highly correlates with fluorescent *in situ* hybridization (FISH) as demonstrated by previous reports (22,23). We found that ERG was expressed in 41.0% of cases, a rate that is in agreement with other previous reports (12,24,25), including a study of the frequency of TMPRSS2-ERG rearrangement in a PCa Southern Brazilian population (26).

Although our findings have shown that ERG-positive cases were associated with lower Gleason score and lower prostate weight, the literature is conflicting and shows varying associations between TMPRSS2-ERG rearrangements and clinicopathological variables. For instance, while one study has shown that patients who expressed ERG fusion protein in prostate tissue (evaluated by FISH) were more prone to present higher Gleason score and PCa-specific death (14), other studies showed lack of association between ERG expression and pathologic parameters (27,28).

Some studies have assessed the importance of the loss of the oncogene PTEN to the prognosis of PCa. By using IHC, Lotan et al. showed that PTEN loss highly correlated with pathologic staging (41% samples with PTEN loss were pT3bN0) and Gleason scores between 8–10 (6). Also, by using IHC, Ahearn et al. (29) found that 25% of PTEN loss in PCa samples were associated with advanced pathologic stage and higher Gleason scores in a population of Caucasian Americans. Our results showed that PTEN loss occurred in 38% of PCa samples with a distribution of homogeneous and heterogeneous pattern close to 50%. Although PTEN loss showed a discrete tendency to be associated with tumor volume (P=0.0659), lymphovascular invasion (P=0.0710), and staging (P=0.0773), these associations did not reach statistical significance. The absence of statistical importance might be explained in part by the small sample size, as well as by heterogeneity of the studied population. Furthermore, false-negative results could also cause misinterpretation of the final count of PTEN loss.

Studies with murine models have suggested the existence of synergy between PTEN loss and ERG contributing to the oncogenesis of PCa (3,30), but in humans, this association is still a matter of debate. In a cohort of RP, Yoshimoto et al. showed that PTEN deletion with the simultaneous presence of TMPRSS2-ERG abnormalities was associated with shorter time to biochemical recurrence of PCa (26). Ahearn et al. (29) evaluated ERG and PTEN by IHC and showed that only the cases with PTEN loss/positive ERG were associated with increased lethality. In the present study, we noted a discrete trend in the frequency of PTEN loss to be higher among those samples with ERG-positive than among ERG-negative samples (48 *vs* 32%). The association of ERG+/PTEN+ samples with lower Gleason score/lower GG found by the present study might indicate a group with a favorable prognosis, but again the absence of clinical follow-up precludes this conclusion.

Diverse molecular subtypes of prostate cancer could contribute to different clinical behaviors and the prevalence of molecular subtypes might vary according to racial and ethnic background (18,19). Thus, Tosoian et al. (31) examined PTEN/ERG status by IHC in self-identified African-Americans (AA) undergoing RP and matched these cases to European-American (EA) patients by pathologic parameters. The rate of PTEN loss was significantly lower in AA compared to EA prostate cancer, similar to the lower rate of ERG expression. Particularly, PTEN loss was observed in 33% ERG-positive tumors, compared to 14% ERG-negative tumors, demonstrating a more than two-fold increase in PTEN loss when ERG expression was present, similar in both groups.

The present study had some limitations. The most relevant was the lack of clinical follow-up information regarding final outcomes, especially biochemical recurrence and disease-specific survival data. Studies evaluating ERG expression in surgically treated patients have shown an association of ERG and longer progression-free survival. It is also important to mention that since it was a single institutional retrospective study, the possibility of bias cannot be excluded.

In summary, we report the frequency of the biomarkers PTEN and ERG in a population of 119 PCa patients from Northeastern Brazil, a population not yet evaluated with these molecular tools. The frequency of ERG expression was 47.5% and PTEN loss 38.1%. Samples with positive ERG expression overlapping with positive PTEN were associated with lower Gleason score and with lower Grade group. The future development of PCa molecular profiling associated with the knowledge of the disease in specific populations could help in stratifying specific risk groups, leading to a more personalized therapeutic decision. Further studies are encouraged in order to integrate molecular studies with clinical information and, in this way, understand how these biomarkers correlate with the overall PCa outcomes.
